# A Novel V2V Cooperative Collision Warning System Using UWB/DR for Intelligent Vehicles

**DOI:** 10.3390/s21103485

**Published:** 2021-05-17

**Authors:** Mingyang Wang, Xinbo Chen, Baobao Jin, Pengyuan Lv, Wei Wang, Yong Shen

**Affiliations:** Institute of Intelligent Vehicles, School of Automotive Studies, Tongji University, No. 4800 Cao’an Highway, Jiading District, Shanghai 201804, China; wangmingyang@sina.cn (M.W.); austin_1@163.com (X.C.); jin_baobao2019@163.com (B.J.); lv_Pengyuan@tongji.edu.cn (P.L.)

**Keywords:** collision warning system, ultra-wideband, dead reckoning, time to collision

## Abstract

The collision warning system (CWS) plays an essential role in vehicle active safety. However, traditional distance-measuring solutions, e.g., millimeter-wave radars, ultrasonic radars, and lidars, fail to reflect vehicles’ relative attitude and motion trends. In this paper, we proposed a vehicle-to-vehicle (V2V) cooperative collision warning system (CCWS) consisting of an ultra-wideband (UWB) relative positioning/directing module and a dead reckoning (DR) module with wheel-speed sensors. Each vehicle has four UWB modules on the body corners and two wheel-speed sensors on the rear wheels in the presented configuration. An over-constrained localization method is proposed to calculate the relative position and orientation with the UWB data more accurately. Vehicle velocities and yaw rates are measured by wheel-speed sensors. An extended Kalman filter (EKF) is applied based on the relative kinematic model to combine the UWB and DR data. Finally, the time to collision (TTC) is estimated based on the predicted vehicle collision position. Furthermore, through UWB signals, vehicles can simultaneously communicate with each other and share information, e.g., velocity, yaw rate, which brings the potential for enhanced real-time performance. Simulation and experimental results show that the proposed method significantly improves the positioning, directing, and velocity estimating accuracy, and the proposed system can efficiently provide collision warning.

## 1. Introduction

The global status report on road safety 2018, launched by the WHO in December 2018, highlighted that the number of annual road traffic deaths had reached 1.35 million [1]. Two-vehicle and multi-vehicle collisions were the most severe types of accidents. Studies showed that more than 80% of road traffic accidents resulted from drivers’ belated responses, and more than 65% resulted in rear-end collisions [2]. Researches indicate that more than 80% of accidents could have been averted if drivers had focused and driven correctly in three seconds before the accident [3].

In recent years, more and more researchers have focused on advanced driving assistance systems (ADAS) to raise consumers’ awareness of safety devices and to reduce the risk of accidents caused by careless driving. As an essential component of the collision warning system, the forward collision warning system (FCWS), can measure the distance with the leading vehicle by itself and warn drivers when the distance between vehicles is less than the safe distance. At present, FCWS using active sensors, such as laser [4,5], radar [6], vision sensor [7,8,9], and infrared [10], has been widely studied. Sanberg et al. [11] presented a stereo vision-based CWS suited for real-time execution in a car. Hernandez et al. designed an object warning collision system for high-conflict vehicle-pedestrian zones using a laser [12]. Coelingh et al. [13,14] proposed a collision avoidance and automatic braking system using a car mounted with radar and camera. Srinivasa et al. [15] proposed an improved CWS combining data from a forward-looking camera and a radar. Although these sensors have high accuracy, they cannot work robustly in bad weather such as rain, snow, and fog and effectively identify dangerous vehicles in visual blind areas. Many advanced algorithms have been proposed to overcome the defects of the sensors [16,17]. However, these algorithms are always limited to particular scenarios, e.g., lane changing [18] and turning [19].

CCWS is an effective solution to this issue, which combines traditional CWS with vehicle-to-infrastructure (V2I) communication and V2V communication [20]. In CCWS, the sensor defects of a single vehicle are supplemented by acquiring information from other vehicles or infrastructures. A V2V-based system shares information among the on-board units (OBU) of vehicles. In V2I systems, accidents and hazardous events are detected by roadside units (RSU) and sent to the OBUs of vehicles [21]. Since vehicles can communicate directly through V2V without dependence on infrastructures, it is more suitable for CWS than V2I. Yang et al. [22] proposed a novel FCWS, which used license plate recognition and vehicle-to-vehicle (V2V) communication to warn the drivers of both vehicles. Xiang et al. [23] proposed an FCWS based on dedicated short-range communication (DSRC) and the global positioning system (GPS). Yang et al. [24] proposed an FCWS combining differential global positioning system (DGPS) and DSRC. Patra et al. [25] proposed a novel FCWS, in which GPS provides the relative positioning information, and vehicles communicate through a vehicular network integrated with smartphones. In general, CCWS can overcome the limitations of the in-vehicle sensor-based CWS by sharing information such as vehicle speed, location, and angle to surrounding vehicles. However, the current V2V based CWSs implement relative positioning and communication separately using different technologies, e.g., predicting collision warning based on radars but communicating through WIFI, which may affect the real-time performance.

To address this issue, the UWB-based CCWS seamlessly combines CWS and V2V without delay. UWB is a communication technology that uses nanosecond narrow pulse signal to transmit data and to measure distances, which has become an effective transmission technology in location-aware sensor networks [26]. Inherently, the UWB-based ranging technology has the advantages of high time resolution and can achieve centimeter-level ranging accuracy [27]. UWB is more adaptable to different environments than traditional sensors used in CWS [28]. There has also been some research on UWB-based CCWS. Sun et al. [29] proposed a UWB/INS (Inertial Navigation System)-based automatic guided vehicle (AGV) collision avoidance system. Liu et al. [30] designed a vehicle collision-avoidance system based on UWB wireless sensor networks. Marianna et al. used UWB to obtain distance information and calculated the collision time to provide collision warnings for workers [31]. Kianfar et al. presented a CWS for the underground mine, which predicted collisions using distances between workers and the mining vehicle measured by UWB [32]. In summary, the existing UWB-based CCWS mainly has two technical routes, which are based on absolute positioning and relative positioning, respectively. The former is hard to popularize due to the small coverage area and high cost of base stations. For the latter, most of the existing research only considers the relative distance between targets rather than the position and ignores the information such as relative velocity and orientation.

To deal with the above problems, a CCWS based on UWB and DR is proposed in this paper. In the proposed system, relative positioning and communication are implemented by UWB simultaneously, which contributes to better real-time performance. Four UWB modules are installed on each vehicle, which makes it possible to calculate not only two-dimension (2D) relative positions but also relative orientations. An over-constrained method is proposed to improve the positioning/directing accuracy. Then, the accuracy and stability of the system are further improved, and the TTC can be estimated with the integration of DR.

This paper is organized as follows: In Section 2, the three subsystems of CCWs are introduced. Section 3 carries on a simulation to evaluate the performance of the system. In Section 4, we conduct experiments and analyze the results. Finally, we summarize the conclusions in Section 5.

## 2. Algorithm and Modeling

The CWS consists of three parts, the UWB-based relative positioning and directing system, the DR system based on wheel-speed sensors, and the TTC estimation system. In the following sections, the UWB-based relative positioning/directing system is shortened to the UWB system. In this section, the UWB and DR subsystems are established. Then, an EKF-based fusion algorithm is proposed to integrate UWB with DR, which significantly improves the accuracy of relative position, orientation, and velocity. Finally, the TTC estimation method in several different collision scenarios is put forward.

### 2.1. The Relative Positioning and Directing System

**Figure 1:**
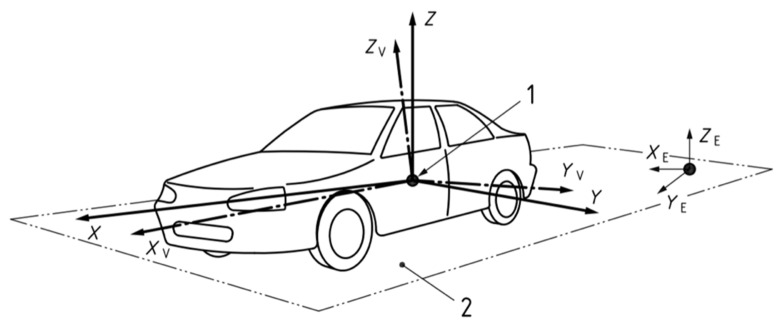
Vehicle axis system.

According to the vehicle axis system regulated by ISO 8855: 2011 [33], as shown in Figure 1, the origin is located at the automotive rear axle center. The X-axis points to the forward of the vehicle, and the Y-axis points to the left. In this paper, all proposed systems are established based on this axis system.

**Figure 2:**
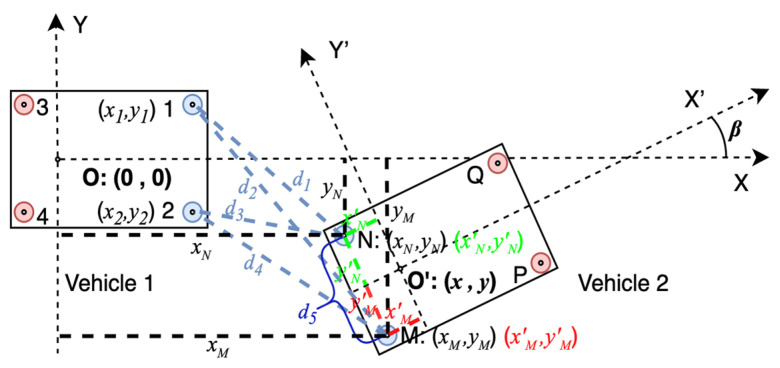
The UWB based relative positioning system model.

Figure 2 shows the UWB system model. XOY represents the coordinate system of vehicle 1. X’O’Y’ represents the coordinate system of vehicle 2. Points 1, 2, 3, and 4 represent the UWB modules on vehicle 1, and points M, N, P, and Q represent the UWB modules on vehicle 2. The coordinate of each UWB module in its own vehicle axis system is known when installed. As Figure 2, $X_{K}={\left[x_{K},y_{K}\right]}^{T}$ is defined as the position of module K in the axis system of vehicle 1 and $X_{K}^{\prime }={\left[x_{K}^{\prime },y_{K}^{\prime }\right]}^{T}$ is defined as the position of module K in the axis system of vehicle 2, where $K=\left(1,\;2,\;3,\;4,\;M,\;N,\;P,\;Q,\;C,O,O^{\prime }\right)$.

With the distances measured by UWB and the coordinates of UWB modules, the relative position and orientation ${\left[x,y,\beta \right]}^{T}$ can be calculated. ${\left[x,y\right]}^{T}$ is the position of vehicle 2 in the axis system of vehicle 1. *β* is the relative orientation, which means the intersection angle of the two vehicles’ driving directions.

As the ranging precision of UWB is very sensitive to NLOS, not all UWB modules are necessary at the same time. Therefore, only four modules, two on each vehicle, in LOS are picked at the same time. The other modules are used to help distinguish multiple solutions. On account of the high time resolution and low multipath effect of UWB signals, it is not complex to distinguish NLOS and LOS signals.

Figure 2 shows a typical driving scenario. Vehicle 2 is changing lanes to the front of vehicle 1. Apparently, rear-end collision risk exists if vehicle 1 drives faster than vehicle 2 and does not brake. Since the CWS is especially necessary in this condition, we take it as an example to interpret our algorithm. In this case, points 1, 2, M, and N are in LOS. Define *d*_1_, *d*_2_, *d*_3_, and *d*_4_ as the real distances shown in Figure 2, and ${\hat{d}}_{1},\;{\hat{d}}_{2},\;{\hat{d}}_{3}$, and ${\hat{d}}_{4}$ as the corresponding measurements ranged by UWB. Other known parameters include $X_{1}={\left[x_{1},y_{1}\right]}^{T}$, $X_{2}={\left[x_{2},y_{2}\right]}^{T}$, $X_{M}^{\prime }={\left[x_{M}^{\prime },y_{M}^{\prime }\right]}^{T}$, $X_{N}^{\prime }={\left[x_{N}^{\prime },y_{N}^{\prime }\right]}^{T}$. Then, we have

(1) $$\{\begin{array}{c} d_{1}=\sqrt{{\left(x_{M}-x_{1}\right)}^{2}+{\left(y_{M}-y_{1}\right)}^{2}}\\ d_{2}=\sqrt{{\left(x_{M}-x_{2}\right)}^{2}+{\left(y_{M}-y_{2}\right)}^{2}}\\ d_{3}=\sqrt{{\left(x_{N}-x_{1}\right)}^{2}+{\left(y_{N}-y_{1}\right)}^{2}}\\ d_{4}=\sqrt{{\left(x_{N}-x_{1}\right)}^{2}+{\left(y_{N}-y_{1}\right)}^{2}} \end{array}.$$

As *d*_1_, *d*_2_, *d*_3_, and *d*_4_ are unknown, ${\hat{d}}_{1},\;{\hat{d}}_{2},\;{\hat{d}}_{3}$, and ${\hat{d}}_{4}$ are substituted into Equation (1) for the estimated positions of M and N, ${\hat{X}}_{M}={\left[{\hat{x}}_{M},{\hat{y}}_{M}\right]}^{T}$ and ${\hat{X}}_{N}={\left[{\hat{x}}_{N},{\hat{y}}_{N}\right]}^{T}$. Then, the estimated distance between M and N can be calculated by Equation (2).

(2) $${\hat{d}}_{5}=\sqrt{{\left({\hat{x}}_{M}-{\hat{x}}_{N}\right)}^{2}+{\left({\hat{y}}_{M}-{\hat{y}}_{N}\right)}^{2}}$$

However, when UWB modules are installed, the real distance between M and N is a determined constant, which can be calculated by Equation (3).

(3) $$d_{5}=\sqrt{{\left(x_{M}^{\prime }-x_{N}^{\prime }\right)}^{2}+{\left(y_{M}^{\prime }-y_{N}^{\prime }\right)}^{2}}$$

When ranging error exists, ${\hat{d}}_{5}\neq d_{5}$. In order to get the least square (LS) solutions that could better meet all the distances, we rewrite Equation (1) as Equation (4).

(4) $$\{\begin{array}{c} d_{1}=\sqrt{{\left(x_{M}-x_{1}\right)}^{2}+{\left(y_{M}-y_{1}\right)}^{2}}\\ d_{2}=\sqrt{{\left(x_{M}-x_{2}\right)}^{2}+{\left(y_{M}-y_{2}\right)}^{2}}\\ d_{3}=\sqrt{{\left(x_{N}-x_{1}\right)}^{2}+{\left(y_{N}-y_{1}\right)}^{2}}\\ d_{4}=\sqrt{{\left(x_{N}-x_{1}\right)}^{2}+{\left(y_{N}-y_{1}\right)}^{2}}\\ d_{5}=\sqrt{{\left(x_{M}-x_{N}\right)}^{2}+{\left(y_{M}-y_{N}\right)}^{2}} \end{array}$$

Significantly, it is an overdetermined nonlinear equation set with five equations and four unknowns. When ranging error exists, the equation set does not have exact solutions. We define function *g* as shown in Equation (5).

(5) $$\begin{array}{ll} g\left(x_{M},y_{M},x_{N},y_{N}\right) & ={\left({\hat{d}}_{1}-\sqrt{{\left(x_{M}-x_{1}\right)}^{2}+{\left(y_{M}-y_{1}\right)}^{2}}\right)}^{2}\\ & +{\left({\hat{d}}_{2}-\sqrt{{\left(x_{M}-x_{2}\right)}^{2}+{\left(y_{M}-y_{2}\right)}^{2}}\right)}^{2}\\ & +{\left({\hat{d}}_{3}-\sqrt{{\left(x_{N}-x_{1}\right)}^{2}+{\left(y_{N}-y_{1}\right)}^{2}}\right)}^{2}\\ & +{\left({\hat{d}}_{4}-\sqrt{{\left(x_{N}-x_{2}\right)}^{2}+{\left(y_{N}-y_{2}\right)}^{2}}\right)}^{2}\\ & +{\left({\hat{d}}_{5}-\sqrt{{\left(x_{M}-x_{N}\right)}^{2}+{\left(y_{M}-y_{N}\right)}^{2}}\right)}^{2} \end{array}$$

Then, the positioning algorithm is converted to an optimization problem with the optimized objective function *g*. According to the first-order necessary condition of optimization problems, the partial derivative of the function *g* should be zero, which is

(6) $$\frac{\partial g}{\partial x_{M}}=\frac{\partial g}{\partial y_{M}}=\frac{\partial g}{\partial x_{N}}=\frac{\partial g}{\partial y_{N}}=0.$$

Several sets of local optimal solutions may be derived from Equation (6). Define $\left[x_{M}^{*},y_{M}^{*},x_{N}^{*},y_{N}^{*}\right]$ as the global LS solution that minimizes the objective function *g*. Then, we have

(7) $${\left[x_{M}^{*},y_{M}^{*},x_{N}^{*},y_{N}^{*}\right]}^{T}=\mathrm{argmin}\left[g\left(x_{M},y_{M},x_{N},y_{N}\right)\right].$$

The solutions of Equation (7) are much more accurate than those of Equation (1). It will be proved later by simulation in Section 3. When no real solutions can be solved from Equation (7), we can go back to Equation (1) for solutions instead.

**Figure 3:**
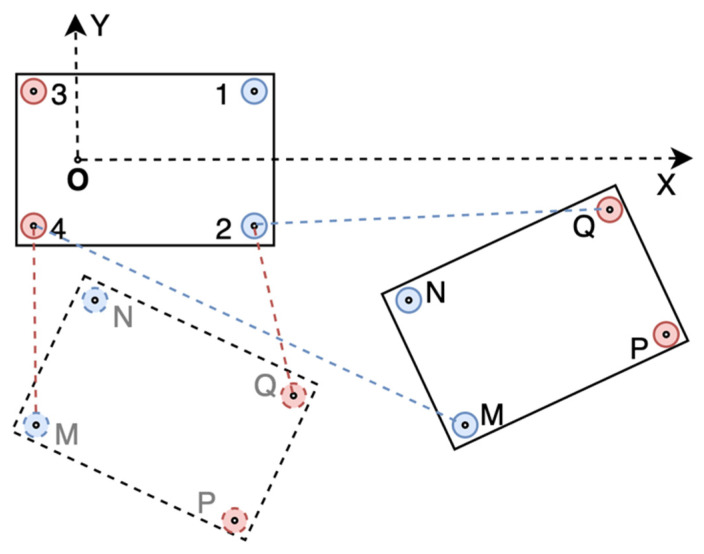
Two sets of solutions.

In the example scenario, we can get two sets of solutions that are symmetric about the line determined by point 1 and point 2, as shown in Figure 3. Dealing with this, ranging information between other UWB modules can be drawn. For example, in Figure 3, distances $\bar{M4}$ and $\bar{Q2}$ can be used to distinguish the two sets of solutions.

After $\left[x_{M}^{*},y_{M}^{*},x_{N}^{*},y_{N}^{*}\right]$ is solved, the relative orientation *β* and position [*x*, *y*]*^T^* can be derived as

(8) $$\begin{array}{c} \beta =\mathrm{atan}2\left(y_{M}-y_{N},x_{M}-x_{N}\right)-\frac{\pi }{2}\\ \left[\begin{array}{c} x\\ y \end{array}\right]=\left[\begin{array}{c} {\bar{x}}^{*}\\ {\bar{y}}^{*} \end{array}\right]-\left[\begin{array}{cc} \cos\left(\beta \right) & -\sin\left(\beta \right)\\ \sin\left(\beta \right) & \cos\left(\beta \right) \end{array}\right]\left[\begin{array}{c} {\bar{x}}^{\prime }\\ {\bar{y}}^{\prime } \end{array}\right] \end{array}$$

where $\left[\begin{array}{c} {\bar{x}}^{*}\\ {\bar{y}}^{*} \end{array}\right]=\frac{1}{2}\left[\begin{array}{c} x_{M}^{*}+x_{N}^{*}\\ y_{M}^{*}+y_{N}^{*} \end{array}\right],\;\left[\begin{array}{c} {\bar{x}}^{\prime }\\ {\bar{y}}^{\prime } \end{array}\right]=\frac{1}{2}\left[\begin{array}{c} x_{M}^{\prime }+x_{N}^{\prime }\\ y_{M}^{\prime }+y_{N}^{\prime } \end{array}\right],\mathrm{atan}2\left(y,x\right)=2\arctan\left(\frac{y}{\sqrt{x^{2}+y^{2}}+x}\right)$.

### 2.2. The DR System Based on Wheel Speed Sensors

**Figure 4:**
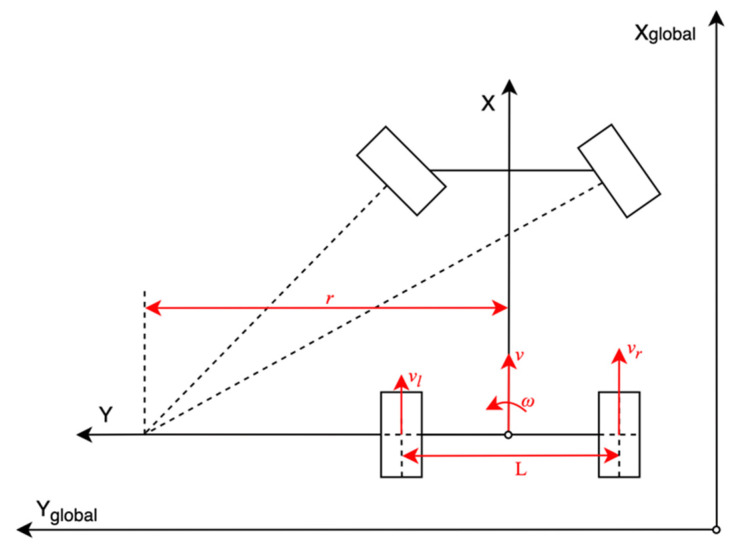
Ackerman steering model.

The proposed system consists of four wheel-speed sensors, which are installed on the rear wheels of two vehicles. According to the Ackerman steering model shown in Figure 4, the instantaneous center of a vehicle is located on the line of the rear axle. The velocity *v*, yaw rate *ω*, and tuning radius *r* can be derived as shown in Equation (9).

(9) $$\{\begin{array}{c} v=\frac{v_{r}+v_{l}}{2}\\ \omega =\frac{v_{r}-v_{l}}{L}\\ r=\frac{v}{\omega } \end{array}$$

where *v_r_* denotes the speed of the right wheel, *v_l_* represents the speed of the left wheel, and *L* indicates the rear wheelbase.

Then, the position ${\left[x_{t+\Delta t},y_{t+\Delta t}\right]}^{T}$ and yaw angle $yaw_{t+\Delta t}$ of the vehicle in the global axis system at time t+Δt can be reckoned by ${\left[x_{t},y_{t}\right]}^{T}$, *v_t_*, and *yam_t_* at time *t* as shown in Equation (10).

(10) $$\left[\begin{array}{c} x_{t+\Delta t}\\ y_{t+\Delta t}\\ yaw_{t+\Delta t} \end{array}\right]=\left[\begin{array}{c} x_{t}+v_{t}\Delta t\cos\left(yaw_{t}\right)\\ y_{t}+v_{t}\Delta t\sin\left(yaw_{t}\right)\\ yaw_{t}+\omega \Delta t \end{array}\right]$$

### 2.3. The EKF Based UWB/DR Fusion Model

We define *X_k_* as the state vector at time *k*. It contains the relative position/orientation $P_{k}={\left[x_{k},y_{k},\beta_{k}\right]}^{T}$, as well as yaw rates and velocities of the two vehicles $S_{k}={\left[\omega_{1_{k}},\omega_{2_{k}},v_{1_{k}},v_{2_{k}}\right]}^{T}$, which can be expressed as Equation (11).

(11) $$X_{k}={\left[x_{k},y_{k},\beta_{k},\omega_{1_{k}},\omega_{2_{k}},v_{1_{k}},v_{2_{k}}\right]}^{T}$$

**Figure 5:**
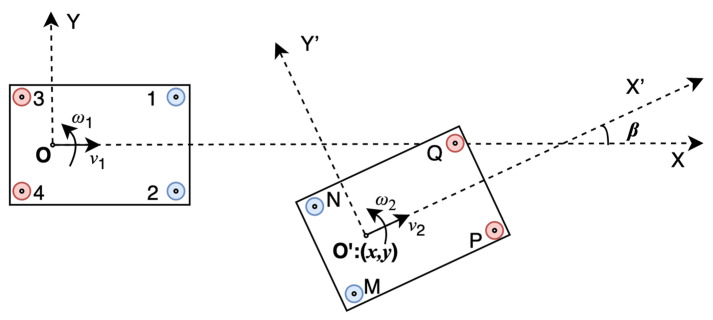
The relative kinematic model.

We define Δ*t* as the time period from time *k* − 1 to time *k*. *X_k_* can be predicted by *X_k_*_−1_ based on the relative kinematics model shown in Figure 5. The state equation can be expressed on the basis of Equation (10) as Equation (12).

(12) $$X_{k}=\left[\begin{array}{c} x_{k}\\ y_{k}\\ \beta_{k}\\ \omega_{1_{k}}\\ \omega_{2_{k}}\\ v_{1_{k}}\\ v_{2_{k}} \end{array}\right]=f\left(X_{k-1},W\right)=\left[\begin{array}{c} C\cos\left(\theta \right)+D\sin\left(\theta \right)\\ -C\sin\left(\theta \right)+D\cos\left(\theta \right)\\ \beta_{k-1}-\theta +\omega_{2_{k-1}}\Delta t+\frac{1}{2}W_{\omega_{2}}\Delta t^{2}\\ \omega_{1_{k-1}}+W_{\omega_{1}}\Delta t\\ \omega_{2_{k-1}}+W_{\omega_{2}}\Delta t\\ v_{1_{k-1}}+W_{v_{1}}\Delta t\\ v_{2_{k-1}}+W_{v_{2}}\Delta t \end{array}\right],$$

where

$C=x_{k-1}-v_{1_{k-1}}t+v_{2_{k-1}}cos\left(\beta_{k-1}\right)\Delta t-W_{v_{1}}\omega \Delta t^{2}/2+W_{v_{2}}cos\left(\beta_{k-1}\right)\Delta t^{2}/2$,

$D=y_{k-1}+v_{2_{k-1}}sin\left(\beta_{k-1}\right)\Delta t+W_{v_{2}}sin\left(\beta_{k-1}\right)\Delta t^{2}/2$,

$\theta =\omega_{1_{k-1}}\Delta t+W_{\omega_{1}}\Delta t^{2}/2$.

Then, the transition matrix of the state vector *A* can be derived as Equation (13).

(13) $$A=\frac{\partial f}{\partial X}=\left[\begin{array}{ccccccc} \cos\left(\theta \right) & \sin\left(\theta \right) & A_{1,3} & A_{1,4} & 0 & -\cos\left(\theta \right)\Delta t & A_{1,7}\\ -\sin\left(\theta \right) & \cos\left(\theta \right) & A_{2,3} & A_{2,4} & 0 & \sin\left(\theta \right)\Delta t & A_{2,7}\\ 0 & 0 & 1 & -\Delta t & \Delta t & 0 & 0\\ 0 & 0 & 0 & 1 & 0 & 0 & 0\\ 0 & 0 & 0 & 0 & 1 & 0 & 0\\ 0 & 0 & 0 & 0 & 0 & 1 & 0\\ 0 & 0 & 0 & 0 & 0 & 0 & 1 \end{array}\right],$$

where

$A_{1,3}=-\cos\left(\theta \right)\sin\left(\beta_{k-1}\right)v_{2_{k-1}}\Delta t+\sin\left(\theta \right)\cos\left(\beta_{k-1}\right)v_{2_{k-1}}\Delta t$,

$A_{1,4}=-Csin\left(\theta \right)\Delta t+Dcos\left(\theta \right)\Delta t$,

$A_{1,7}=\cos\left(\theta \right)\cos\left(\beta_{k-1}\right)\Delta t+\sin\left(\theta \right)\sin\left(\beta_{k-1}\right)\Delta t$,

$A_{2,3}=\sin\left(\theta \right)\sin\left(\beta_{k-1}\right)v_{2_{k-1}}\Delta t+\cos\left(\theta \right)\cos\left(\beta_{k-1}\right)v_{2_{k-1}}\Delta t$,

$A_{2,4}=-C\cos\left(\theta \right)\Delta t-D\sin\left(\theta \right)\Delta t$,

$A_{2,7}=-\sin\left(\theta \right)\cos\left(\beta_{k-1}\right)\Delta t+\cos\left(\theta \right)\sin\left(\beta_{k-1}\right)\Delta t$.

Similarly, the transition matrix of process noise is:

(14) $$G=\frac{\partial f}{\partial W}=\left[\begin{array}{cccc} G_{1,1} & 0 & G_{1,3} & G_{1,4}\\ G_{2,1} & 0 & G_{2,3} & G_{2,4}\\ -\Delta t^{2}/2 & \Delta t^{2}/2 & 0 & 0\\ \Delta t & 0 & 0 & 0\\ 0 & \Delta t & 0 & 0\\ 0 & 0 & \Delta t & 0\\ 0 & 0 & 0 & \Delta t \end{array}\right],$$

where

$G_{1,1}=\left[-C\sin\left(\theta \right)+D\cos\left(\theta \right)\right]\Delta t^{2}/2$,

$G_{1,3}=-\cos\left(\theta \right)\Delta t^{2}/2$,

$G_{1,4}=\left[\cos\left(\theta \right)\cos\left(\beta_{k-1}\right)+\sin\left(\theta \right)\sin\left(\beta_{k-1}\right)\right]\Delta t^{2}/2$,

$G_{2,1}=\left[-C\cos\left(\theta \right)-D\sin\left(\theta \right)\right]\Delta t^{2}/2$,

$G_{2,3}=\sin\left(\theta \right)\Delta t^{2}/2$,

$G_{2,4}=[-\sin\left(\theta \right)\cos\left(\beta_{k-1}\right)+\cos\left(\theta \right)\sin\left(\beta_{k-1}\right)]\;\Delta t^{2}/2$.

The error covariance matrix *Q* of process noise consists of error covariances of speeds and yaw rates, that is:

(15) $$Q=\mathrm{cov}\left(W\right)=\left[\begin{array}{cccc} \sigma_{\omega_{1}}^{2} & 0 & 0 & 0\\ 0 & \sigma_{\omega_{2}}^{2} & 0 & 0\\ 0 & 0 & \sigma_{v_{1}}^{2} & 0\\ 0 & 0 & 0 & \sigma_{v_{2}}^{2} \end{array}\right].$$

Thus, the predicting process of the model is:

(16) $$\begin{array}{c} {\hat{X}}_{k}^{-}=f\left({\hat{X}}_{k-1}\right)\\ P_{k}^{-}=AP_{k-1}A+GQG. \end{array}$$

We define *Z_k_* as the observation vector, containing the relative position and orientation of vehicle 2 measured by the UWB system, four wheel-speeds measured by the DR system, and the observation noise *V_k_*. Then, the observation equation can be expressed as Equation (17).

(17) $$Z_{k}=\left[\begin{array}{c} x_{UWB,k}\\ y_{UWB,k}\\ \beta_{UWB,k}\\ v_{r1,k}\\ v_{l1,k}\\ v_{r2,k}\\ v_{l2,k} \end{array}\right]=HX_{k}+V_{k}$$

Referring to Equation (9), the velocities and yaw rates of the two vehicles can be expressed by the velocities measured by wheel-speed sensors as Equation (18).

(18) $$\left[\begin{array}{c} v_{1r}\\ v_{1l}\\ v_{2r}\\ v_{2l} \end{array}\right]=\left[\begin{array}{cccc} L_{1}/2 & 0 & 1 & 0\\ -L_{1}/2 & 0 & 1 & 0\\ 0 & L_{2}/2\; & 0 & 1\\ 0 & -L_{2}/2 & 0 & 1 \end{array}\right]\left[\begin{array}{c} \omega_{1}\\ \omega_{2}\\ v_{1}\\ v_{2} \end{array}\right]$$

Then, the Jacobian matrix *H* is obtained as Equation (19).

(19) $$H=\left[\begin{array}{ccccccc} 1 & 0 & 0 & 0 & 0 & 0 & 0\\ 0 & 1 & 0 & 0 & 0 & 0 & 0\\ 0 & 0 & 1 & 0 & 0 & 0 & 0\\ 0 & 0 & 0 & L_{1}/2 & 0 & 1 & 0\\ 0 & 0 & 0 & -L_{1}/2 & 0 & 1 & 0\\ 0 & 0 & 0 & 0 & L_{2}/2 & 0 & 1\\ 0 & 0 & 0 & 0 & -L_{2}/2 & 0 & 1 \end{array}\right]$$

The estimating process is:

(20) $$\begin{array}{c} K_{k}=P_{k}^{-}H^{T}{\left(HP_{k}^{-}H^{T}+R\right)}^{-1}\\ X^{-}={\hat{X}}_{k}^{-}+K_{k}\left(Z_{k}-H{\hat{X}}_{k}^{-}\right)\\ P_{k}=P_{k}^{-}-K_{k}HP_{k}^{-}. \end{array}$$

In Equation (20), *R* represents the error covariance matrix of *Z_k_*. It can be divided into the error covariance matrix of the UWB system *R_UWB_* and the error covariance matrix of the DR system *R_DR_*. That is:

(21) $$R=\mathrm{cov}\left(V_{k}\right)=\left[\begin{array}{cc} R_{UWB} & 0\\ 0 & R_{DR} \end{array}\right],$$

where $R_{UWB}=\left[\begin{array}{ccc} \sigma_{x}^{2} & 0 & 0\\ 0 & \sigma_{y}^{2} & 0\\ 0 & 0 & \sigma_{\beta }^{2} \end{array}\right]$, $R_{DR}=\left[\begin{array}{cccc} \sigma_{v_{r1}}^{2} & 0 & 0 & 0\\ 0 & \sigma_{v_{l1}}^{2} & 0 & 0\\ 0 & 0 & \sigma_{v_{r2}}^{2} & 0\\ 0 & 0 & 0 & \sigma_{v_{l2}}^{2} \end{array}\right]$.

*R_DR_* is decided by measurement errors of the wheel-speed sensors directly, whereas *R_UWB_* is decided by positioning and directing errors, which is indirectly decided by the ranging error of UWB modules. Define *D* = [*d*_1_, *d*_2_, *d*_3_, *d*_4_]. On the basis of Equation (5), we can derive the relationship between the deviation *D* and the deviation of UWB modules’ position *X_M_* and *X_N_* as Equation (22). *d*_5_ is ignored because it is not a measurement but a constant, which means $dd_{5}=0$.

(22) $$dD=\left[\begin{array}{c} dd_{1}\\ dd_{2}\\ dd_{3}\\ dd_{4} \end{array}\right]=\frac{\partial D}{\partial \left(x_{M},y_{M},x_{N},y_{N}\right)}\left[\begin{array}{c} dx_{M}\\ dy_{M}\\ dx_{N}\\ dy_{N} \end{array}\right]=F_{D}\left[\begin{array}{c} dx_{M}\\ dy_{M}\\ dx_{N}\\ dy_{N} \end{array}\right],$$

*F_D_* can be derived as Equation (23).

(23) $$F_{D}=\left[\begin{array}{cccc} \frac{x_{M}-x_{1}}{d_{1}} & \frac{y_{M}-y_{1}}{d_{1}} & 0 & 0\\ \frac{x_{M}-x_{2}}{d_{2}} & \frac{y_{M}-y_{2}}{d_{2}} & 0 & 0\\ 0 & 0 & \frac{x_{N}-x_{1}}{d_{3}} & \frac{y_{N}-y_{1}}{d_{3}}\\ 0 & 0 & \frac{x_{N}-x_{2}}{d_{4}} & \frac{y_{N}-y_{2}}{d_{4}} \end{array}\right].$$

where $d_{1}={\hat{d}}_{1}$, $d_{2}={\hat{d}}_{2}$, $d_{3}={\hat{d}}_{3}$, $d_{4}={\hat{d}}_{4}$, $x_{M}=x_{M}^{*}$, $y_{M}=y_{M}^{*}$,$\;x_{N}=x_{N}^{*}$,$\;y_{N}=y_{N}^{*}$.

From Equation (8), we can get the relationship between the deviation of the vehicle position and orientation *X_UWB_* = [*x*, *y*, *β*] and the deviation of the UWB modules’ position *X_M_* and *X_N_* as Equation (24).

(24) $$dX_{UWB}=\left[\begin{array}{c} x\\ y\\ \beta \end{array}\right]=\frac{\partial X_{UWB}}{\partial \left(x_{M},y_{M},x_{N},y_{N}\right)}\left[\begin{array}{c} dx_{M}\\ dy_{M}\\ dx_{N}\\ dy_{N} \end{array}\right]=F_{X_{UWB}}\left[\begin{array}{c} dx_{M}\\ dy_{M}\\ dx_{N}\\ dy_{N} \end{array}\right].$$

$F_{X_{UWB}}$ can be derived as Equation (25).

(25) $$F_{X_{UWB}}=\left[\begin{array}{cccc} F_{1,1} & F_{1,2} & F_{1,3} & F_{1,4}\\ F_{2,1} & F_{2,2} & F_{2,3} & F_{2,4}\\ -\frac{\left(y_{M}-y_{N}\right)}{d_{5}^{2}} & \frac{\left(x_{M}-x_{N}\right)}{d_{5}^{2}} & \frac{\left(y_{M}-y_{N}\right)}{d_{5}^{2}} & -\frac{\left(x_{M}-x_{N}\right)}{d_{5}^{2}} \end{array}\right],$$

where

$F_{1,1}=1/2+\left[x_{M}^{\prime }\cos\left(\beta \right)-y_{M}^{\prime }\sin\left(\beta \right)\right]\left(y_{M}-y_{N}\right)/d_{5}^{2}$,

$F_{1,2}=-\left[x_{M}^{\prime }\cos\left(\beta \right)-y_{M}^{\prime }\sin\left(\beta \right)\right]\left(x_{M}-x_{N}\right)/d_{5}^{2}$,

$F_{1,3}=1/2-\left[x_{M}^{\prime }\cos\left(\beta \right)-y_{M}^{\prime }\sin\left(\beta \right)\right]\left(y_{M}-y_{N}\right)/d_{5}^{2}$,

$F_{1,4}=\left[x_{M}^{\prime }\cos\left(\beta \right)-y_{M}^{\prime }\sin\left(\beta \right)\right]\left(x_{M}-x_{N}\right)/d_{5}^{2}$,

$F_{2,1}=\left[y_{M}^{\prime }\cos\left(\beta \right)+x_{M}^{\prime }\sin\left(\beta \right)\right]\left(y_{M}-y_{N}\right)/d_{5}^{2}$,

$F_{2,2}=1/2-\left[y_{M}^{\prime }\cos\left(\beta \right)+x_{M}^{\prime }\sin\left(\beta \right)\right]\left(x_{M}-x_{N}\right)/d_{5}^{2}$,

$F_{2,3}=-\left[y_{M}^{\prime }\cos\left(\beta \right)+x_{M}^{\prime }\sin\left(\beta \right)\right]\left(y_{M}-y_{N}\right)/d_{5}^{2}$,

$F_{2,4}=1/2+\left[y_{M}^{\prime }\cos\left(\beta \right)+x_{M}^{\prime }\sin\left(\beta \right)\right]\left(x_{M}-x_{N}\right)/d_{5}^{2}$,

$x_{MN}^{\prime }=\left(x_{M}^{\prime }+x_{N}^{\prime }\right)/2,\;y_{MN}^{\prime }=\left(y_{M}^{\prime }+y_{N}^{\prime }\right)/2$,

$x_{M}=x_{M}^{*},\;y_{M}=y_{M}^{*},\;x_{N}=x_{N}^{*},\;y_{N}=y_{N}^{*}$.

Then, *R_UWB_* can be expressed as Equation (26).

(26) $$R_{UWB}=F_{X_{UWB}}{\left(F_{D}^{T}F_{D}\right)}^{-1}F_{D}^{T}R_{D}F_{D}{\left(F_{D}^{T}F_{D}\right)}^{-1}F_{X_{UWB}}^{T},$$

where $R_{D}=\mathrm{diag}\left(\sigma_{d_{1}}^{2},\sigma_{d_{2}}^{2},\sigma_{d_{3}}^{2},\sigma_{d_{4}}^{2},\sigma_{d_{5}}^{2}\right)$ is determined directly by UWB ranging error covariance.

### 2.4. The Collision Warning Model

CWS mainly works in two ways, headway measurement warning (HMW) and TTC-based warning [34]. Both of them need to measure the distance to the front vehicle but estimate the collision time with different speeds as Equation (27).

(27) $$\begin{array}{c} Headway\;Collision\;Time=\frac{Headway}{v_{RearVehicle}}\\ TTC=\frac{Headway}{v_{RearVehicle}-v_{FrontVehicle}} \end{array}$$

The TTC-based system takes relative velocity into account, so it provides a more accurate collision warning. In this paper, the proposed system allows vehicles to share information through UWB, such as velocities. The TTC method is apparently the better choice.

**Figure 6:**
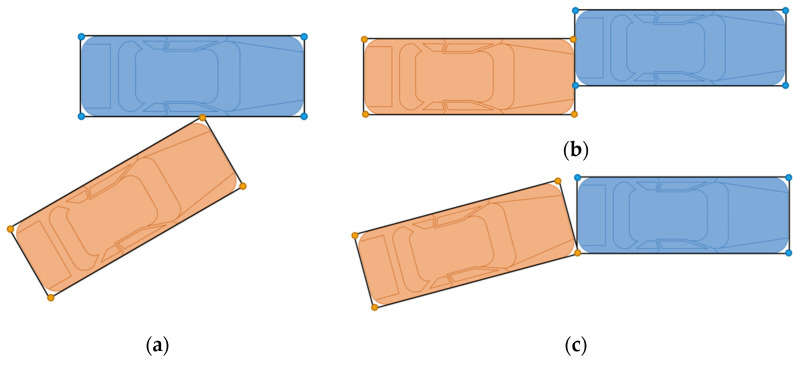
Collision types. (**a**) Point-to-edge collision; (**b**) Edge-to-edge collision; (**c**) Point-to-point collision.

Two vehicles driving on the road have the probability of collisions in various types, such as head-to-head collision, rear-end collision, and side collision. Different kinds of collisions may happen at different times. That means all cases need to be taken into account in order to obtain the exact TTC. Before establishing the collision warning model, we simplified the shape of a vehicle as a rectangle. With this assumption, all kinds of collisions can be described as point-to-edge collisions. Edge-to-edges collisions and point-to-point collisions are also covered by point-to-edge collisions, as shown in Figure 6.

**Figure 7:**
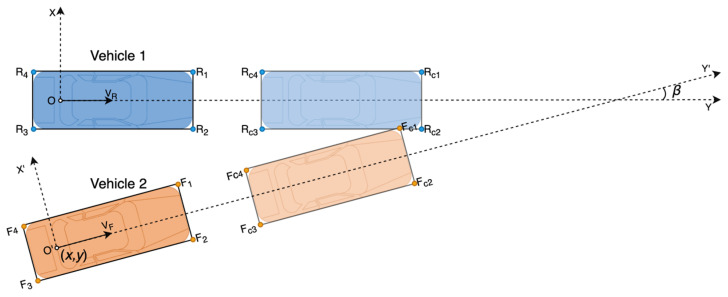
The collision warning model.

After unifying different collision types, TTC can be calculated in the same way. We take the collision type shown in Figure 7 as an example. In this case, the front left corner of vehicle 2 collides on the right edge of vehicle 1. As we defined in Section 2.1, the coordinate of a point in the axis system of vehicle 1 is expressed as $X_{k}={\left[x_{k},y_{k}\right]}^{T}$, and ${X_{k}}^{\prime }={\left[{x_{k}}^{\prime },{y_{k}}^{\prime }\right]}^{T}$ in the axis system of vehicle 2. *R_i_* (*i* = 1,2,3,4) represents the four corners of vehicle 1. *F_i_* (*i* = 1,2,3,4) represents the four corners of vehicle 2. Therefore, the coordinate of $R_{i}$ is $X_{R_{i}}={\left[x_{R_{i}},y_{R_{i}}\right]}^{T}$, which is known by measuring the size of the vehicle 1. Similarly, $X_{F_{i}}^{\prime }={\left[x_{F_{i}}^{\prime },y_{F_{i}}^{\prime }\right]}^{T}$ is also known by measuring the size of vehicle 2. The relative position of *X* = [*x*, *y*]*^T^* and the relative orientation *β* are estimated by the UWB/DR system. Then, the coordinates of vehicle 2′s corners in the axis system of vehicle 1 can be derived as Equation (28).

(28) $$\left[X_{F_{1}},X_{F_{2}},X_{F_{3}},X_{F_{4}}\right]=R\left[X_{F_{1}}^{\prime },X_{F_{2}}^{\prime },X_{F_{3}}^{\prime },X_{F_{4}}^{\prime }\right]+X\left[1,1,1,1\right]$$

where $R=\left[\begin{array}{cc} \cos\left(\beta \right) & -\sin\left(\beta \right)\\ \sin\left(\beta \right) & \cos\left(\beta \right) \end{array}\right]$.

We define all the points at the collision time as $R_{C_{i}}$ and $F_{C_{i}}$, and their coordinates as $X_{R_{C_{i}}}={\left[x_{R_{C_{i}}},y_{R_{C_{i}}}\right]}^{T}$, $X_{F_{C_{i}}}={\left[x_{F_{C_{i}}},y_{F_{C_{i}}}\right]}^{T}$. The velocity vectors of the two vehicles are known for the UWB/DR system, which are $V_{R}={\left[v_{R}\cos\left(\beta_{R}\right),v_{R}\cos\left(\beta_{R}\right)\right]}^{T}\;\left(\beta_{R}=0\right)$ and $V_{F}={\left[v_{F}\cos\left(\beta_{F}\right),v_{F}\sin\left(\beta_{F}\right)\right]}^{T}\;\left(\beta_{F}=\beta \right)$. Assume that point $F_{i}$ collides on the edge between $R_{j}$ and *R_k_* at time $t_{F_{i},R_{jk}}$. Then $X_{F_{C_{i}}}$, $X_{R_{C_{j}}}$, and $X_{R_{C_{k}}}$ can be expressed as Equation (29).

(29) $$\begin{array}{c} X_{F_{C_{i}}}=X_{F_{i}}+V_{F}t_{F_{i},R_{jk}}\\ X_{R_{C_{j}}}=X_{R_{j}}+V_{R}t_{F_{i},R_{jk}}\\ X_{R_{C_{k}}}=X_{R_{k}}+V_{R}t_{F_{i},R_{jk}} \end{array}$$

Point *F_i_* collides on the edge between *R_j_* and *R_k_* means $F_{c_{i}}$ is on the segment $\bar{R_{C_{j}}R_{C_{k}}}$, which can be expressed as Equation (30).

(30) $$\vec{F_{C_{i}}R_{C_{j}}}\cdot \vec{F_{C_{i}}R_{C_{k}}}=-\|\vec{F_{C_{i}}R_{C_{j}}}\|\|\vec{F_{C_{i}}R_{C_{k}}}\|$$

Solution *t* of Equation (30) is the collision time under the condition that corners of vehicle 2 collide on edges of vehicle 1, including 16 different conditions altogether. In the other 16 cases in which the corners of vehicle 1 collide on the edges of vehicle 2, the collision times can be calculated similarly. Thirty-two collision times can be calculated in total. Ignoring negative values, the minimum of the rest value is TCC. That is:

(31) $$\begin{array}{c} TTC=\min\left(t_{R_{i},F_{jk}},t_{F_{i},R_{jk}}\right),\;\left(i=1,2,3,4;jk=12,23,34,41\right),\\ t_{R_{i},F_{jk}}\geq 0,t_{F_{i},R_{jk}}\geq 0. \end{array}$$

When *TTC* → ∞ or *TTC* < 0, there is no risk of collision.

## 3. Simulation

In this section, simulation is conducted to evaluate our algorithm. Firstly, the accuracy of the UWB positioning and directing system is validated by comparing the algorithm with and without the constraint of *d*_5_. Secondly, the accuracy of the UWB/DR fusion model based on EKF is compared to the accuracy of UWB and DR separately. Finally, plenty of driving scenarios are generated to evaluate the success rate of the CWS.

### 3.1. Simulation of the Overconstrained UWB Positioning and Directing System

**Figure 8:**
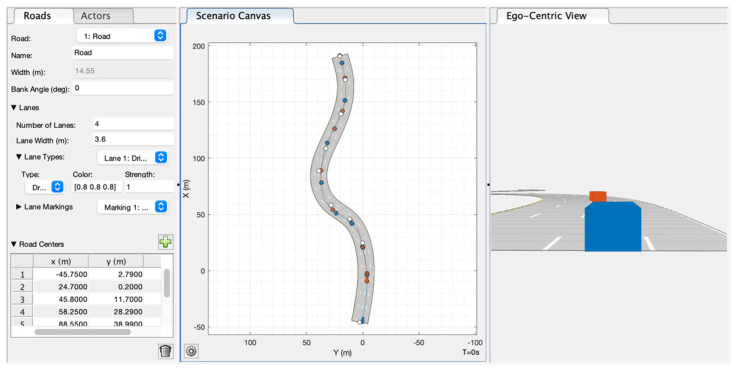
The virtual scenario in the driving scenario designer.

In Section 2.1, a relative positioning/directing algorithm with the constraint of $d_{5}$ is proposed. Its performance is simulated in this section. Firstly, a driving scenario is established in the driving scenario designer of MATLAB as shown in Figure 8. The blue cube represents vehicle 1, and the red cube represents vehicle 2. The lines in blue and red denote their driving track. Kinematic parameters of vehicle and positions of UWB modules and wheel sensors in their own vehicle axis system are defined in the model. The UWB ranging error is set to *σ_d_* = 0.05 m, and the wheel speed error is set to *σ_v_* = 0.2 m/s referring to the sensors we will use in experiments. Calculating results of our algorithm are compared to the real values exported by the model.

**Figure 9:**
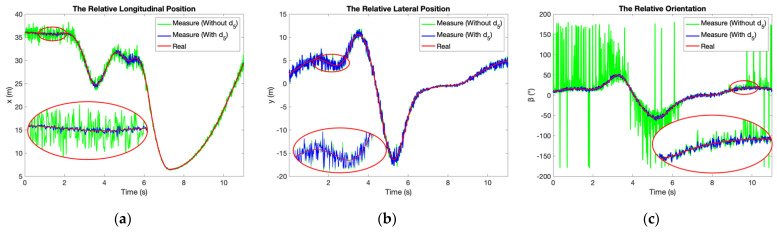
Comparison of relative positioning and directing algorithm with and without the constraint of *d*_5_: (**a**) The relative longitudinal position *x*; (**b**) The relative lateral position *y*; (**c**) The relative orientation *β*.

Solutions of the algorithm with and without the constraint of *d*_5_ are compared in Figure 9 and Table 1. The improvement of accuracy with the derivation of *d*_5_ is very intuitive, especially for *x* and *β*. In Table 1, the root mean square error (RMSE) is recommended to compare their accuracy quantitatively.

### 3.2. Simulation of the UWB/DR Fusion Algorithm

**Figure 10:**
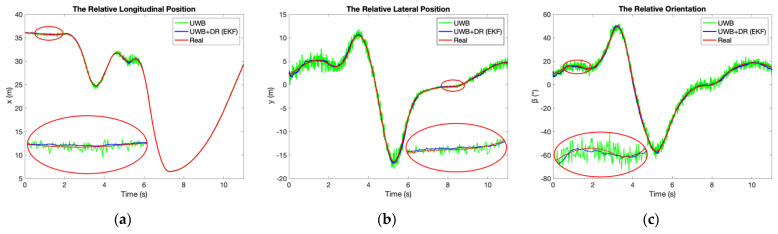
Comparison of positioning and directing performance using UWB and fusion of UWB/DR: (**a**) The relative longitudinal position *x*; (**b**) The relative lateral position *y*; (**c**) The relative orientation *β*.

We also take the scenario in Section 3.1 as an example to validate the performance of the UWB/DR fusion algorithm. The comparison results are shown in Figure 10 and Table 2. The proposed UWB/DR fusion method based on EKF significantly improves the accuracy and stability of positioning and directing.

**Figure 11:**
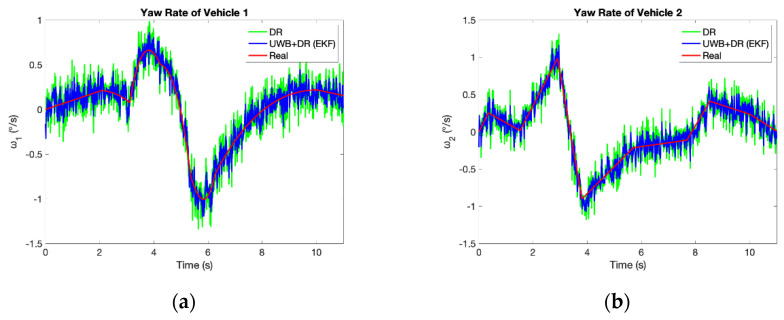
Comparison of yaw rates measured by DR and estimated by UWB/DR: (**a**) Yaw rate of vehicle 1; (**b**) Yaw rate of vehicle 2.

**Figure 12:**
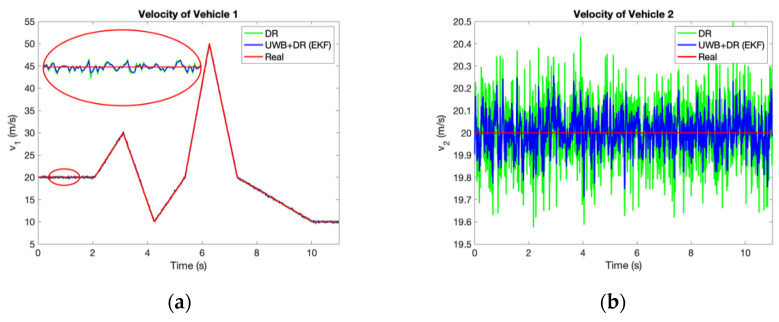
Comparison of velocities measured by DR and estimated by UWB/DR: (**a**) Velocity of vehicle 1; (**b**) Velocity of vehicle 2.

Figure 11 and Figure 12 and Table 3 compare the accuracy of yaw rates and velocities estimated by UWB/DR to DR. They are improved significantly as well, which contributes to the better prediction accuracy of TTC in the next section.

### 3.3. Simulation of CWS based on TTC Estimation

**Figure 13:**
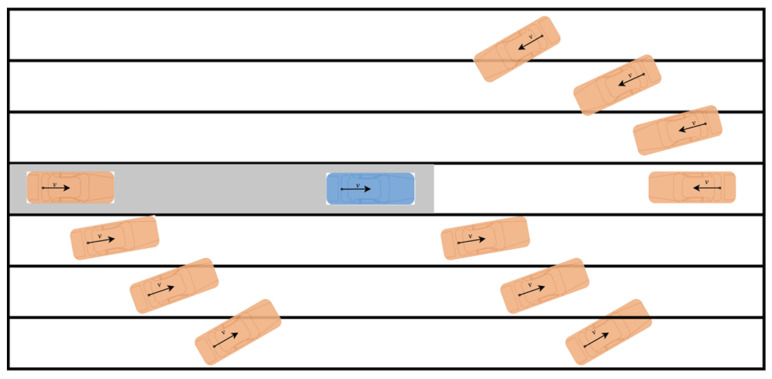
TTC simulating scenarios.

In this section, we generate plenty of driving scenarios with different velocities, relative positions, and relative orientations, as shown in Figure 13. The ranges of parameters are set as outlined in Table 4.

**Figure 14:**
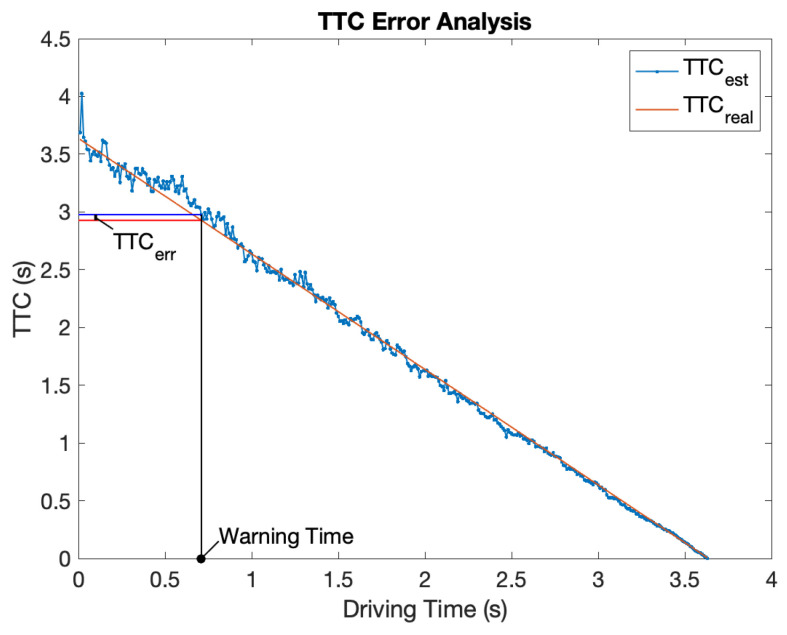
TTC estimation error.

*TTC_real_* is certain when a scenario is established, and *TTC_est_* estimated by CWS is calculated every 10 ms. The collision warning threshold is set to 3.0 s. It means that when *TTC_est_* ≤ 3.0 s, the CWS will send an alert. *TTC_err_* = *TTC_est_* – *TTC_real_* denotes the TTC error at the warning time as Figure 14.

In order to guarantee driving safety, we set 2.7 s as the latest warning time. If the system does not work when the vehicle is colliding within 2.7 s, the collision warning evaluation is failed. In addition, in order not to disturb the driver too much, if the system sends alerts when vehicles have no risk of collision within 4 s, we regard the warning as false. Then, *TTC_err_* can be divided into three conditions corresponding to three evaluations of collision warning as Equation (32).

(32) $$TTC_{err}\{\begin{array}{cc} >0.3 & \mathrm{Failed}\\ \in \left[-1,0.3\right] & \mathrm{Correct}\\ <-1 & \mathrm{False} \end{array}$$

- “Failed” denotes warning too late or not warning;
- “Correct” denotes warning in the proper time period;
- “False” denotes warning too early or warning by mistake.

The scenario marked with gray background is the typical rear-end collision scenario, which is the most critical function of a collision warning system. One hundred and ninety-six rear-end collision scenarios are generated, and Table 5 shows the results. In all the 196 simulation scenarios, two of them behave false, which means that the collision warning is triggered too early. All of the others perform correctly. It shows the reliability of the proposed CWS in the most common rear-end collision scenarios.

Then, we emulate other scenarios in which two vehicles drive in any lanes from any positions to any directions defined in Figure 13 and Table 4. Results are shown in Table 6. The scenarios with initial *TTC_real_* less than 3 s will not be considered. In the remaining 10,823 scenarios, 10,593 of them perform correctly. The collision warning success rate is 97.9%.

## 4. Experiments

In this section, experiments are divided into two parts: straight driving experiments and curved driving experiments. The straight driving experiments are conducted referring to JT/T883-2014, which describes the standard experiments for FCWS, published by the Ministry of Transport of the People’s Republic of China (MOT). As JT/T883-2014 only regulates straight driving experiments, to further validate the performance of our system, curved driving experiments are conducted in addition. Since the CWS is implemented based on the UWB/DR relative positioning/directing system, the positioning/directing accuracy can reflect the performance of the CWS. Therefore, in the curved experiments, we drive through complex routes and compare the positioning/directing accuracy to the parameters of a commercial millimeter-wave radar (MMWR) used for collision warning.

### 4.1. Experimental Equipment and Environment

**Figure 15:**
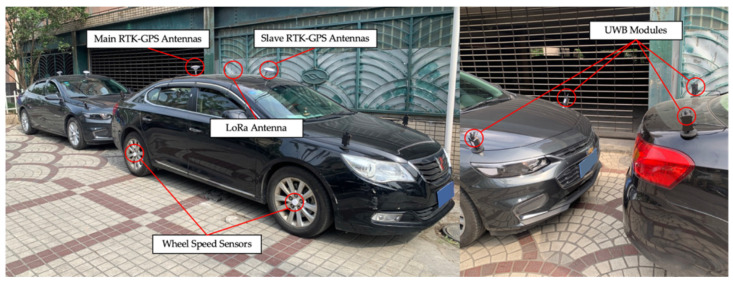
Experimental Equipment.

Figure 15 shows the equipment used in the experiments. Two vehicles are required in the experiments for relative positioning and directing. UWB modules are installed on the corners of the vehicles. Four wheel-speed sensors designed by our team are installed on the centers of the wheels. The wheel-speed measurements are transmitted to a receiver inside the vehicle wirelessly, which receives the velocity information from the four wheels and then sends it to the controller area network (CAN) bus. In the proposed system, only the speeds of the rear wheels are used. UWB modules are also developed by our team based on DW1000. Two vehicles share data through UWB. All data are transferred to the CAN bus and recorded by the computer using a USB-CAN adapter. A computing terminal receives sensor data from the CAN bus and calculates the relative position, direction, velocity, and TTC. Results from the computing terminal are compared to the measurement of a high-precision integrated positioning system, which combines dual-antenna real-time kinematic (RTK)-GPS and INS. The long-range radio (LoRa) antenna is used to receive differential signals from the RTK-GPS base station, which is installed in the testing ground. A total station is used to measure the relative coordinates of the UWB modules to the main RTK-GPS antenna. It should be noted that the main GPS antenna is not right above the center of the rear wheels. The deviation needs to be derived from measurements of the total station and compensated in the algorithm.

**Figure 16:**
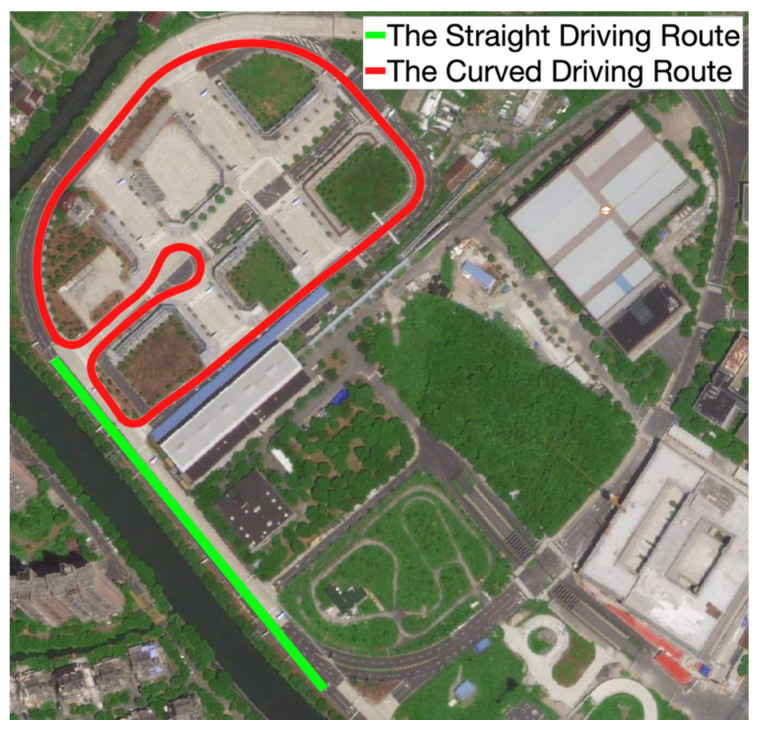
The testing ground and vehicle driving routes.

Figure 16 shows the testing ground in which we conduct experiments. The driving routes of the two types of experiments are also marked in Figure 16.

### 4.2. Straight Driving Experiments

**Figure 17:**
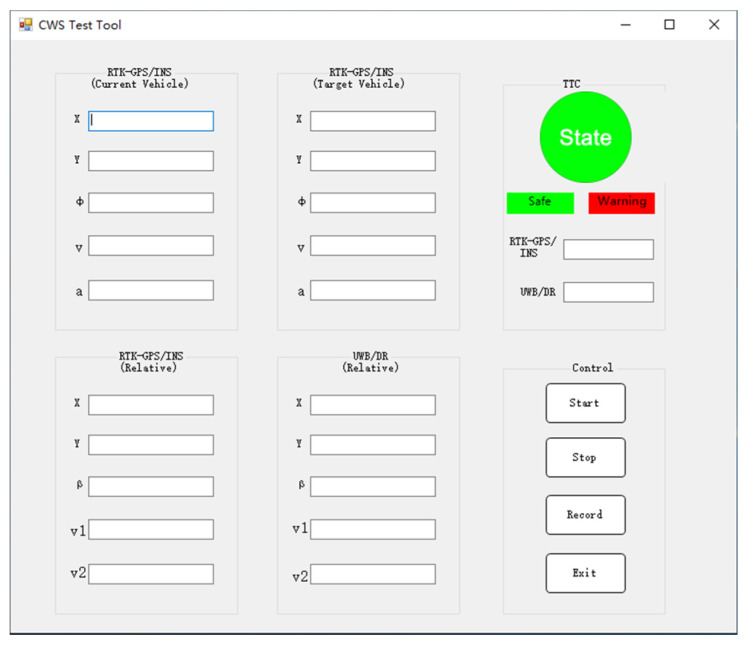
Vehicle state display software.

According to JT/T883-2014 [35], experiments for FCWS consist of three tests. Each test needs repeating seven times. Only if five of them were passed, and no two consecutive failed tests exist could the test be evaluated as passed. In the standard experiments, the headway distances, velocities, and accelerations of vehicles need controlling around specific values, so we design software as shown in Figure 17, with necessary parameters displayed, which helps drivers better control vehicles and records necessary data. The TTC derived from the data of the RTK-GPS/INS is recognized as real TTC.

#### 4.2.1. Test 1

**Figure 18:**
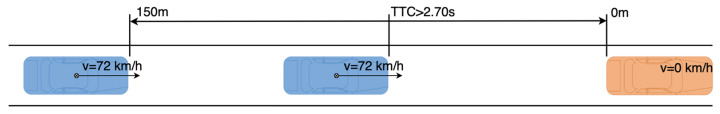
Test 1 in the straight driving experiments.

Test 1 is designed as shown in Figure 18. The rear vehicle drives at the speed of 72 km/h toward the parked front vehicle from an inertial headway distance of 150 m. If the collision warning system is triggered before the real TTC is 2.7 s, the test is passed. Otherwise, the test is failed.

#### 4.2.2. Test 2

**Figure 19:**
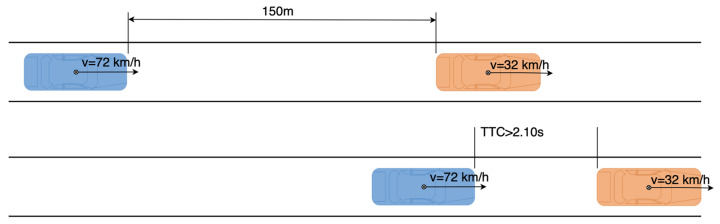
Test 2 in the straight driving experiments.

Test 2 is designed as shown in Figure 19. The rear vehicle drives at the speed of 72 km/h toward the front vehicle, which drives at the speed of 32 km/h, from an initial headway distance of 150 m. If the collision warning system is triggered before the real TTC is 2.1 s, the test is passed. Otherwise, the test is failed.

#### 4.2.3. Test 3

**Figure 20:**
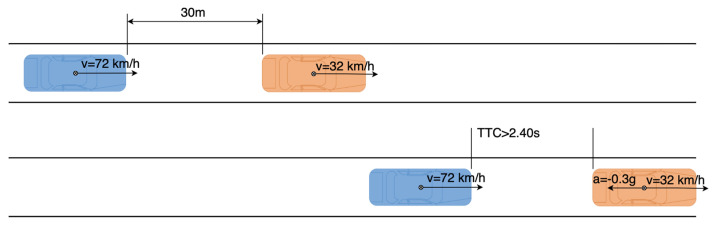
Test 3 in the straight driving experiments.

Test 3 is designed as Figure 20. The rear vehicle drives at the speed of 72 km/h toward the front vehicle, which drives at the speed of 32 km/h and decelerates with the acceleration of −0.3 g. If the collision warning system is triggered before the real TTC is 2.4 s, the test is passed. Otherwise, the test is failed.

#### 4.2.4. Results Analysis of the Straight Driving Experiments

During each test, two TTC values are calculated: (1) *TTC_real_*, which is derived from the RTK-GPS/INS information; (2) *TTC_CWS_*, which is estimated using the UWB/DR measurements. Since the terminating conditions in the three experiments are different, to satisfy all the three tests and reserve some margin, we set the warning *TTC_CWS_* to 3.0 s. The software in Figure 17 will send a warning when either *TTC_real_* or *TTC_CWS_* reaches its marginal value. If *TTC_real_* reaches the regulated marginal value when *TTC_CWS_* is still greater than 3.0 s, the test is terminated and evaluated as failed. In the standard, only the minimum threshold of the collision warning time is regulated, whereas the maximum threshold is not. In other words, the standard only cares about “how safe” the warning is, with no consideration of “how accurate” it is. However, as we explained in Section 3, too early warnings are annoying and offensive, so we set 4.0 s as the upper limit. If the CWS is triggered when *TTC_real_* > 4.0 s, we also regard the test as failed. According to JT/T883-2014, each test needs repeating seven times. Table 7, Table 8 and Table 9 show the results of the three tests, respectively.

According to Table 7, Table 8 and Table 9, all the tests were passed, which proves that the proposed system can satisfy the requirement of MOT and has the ability to provide collision warning for vehicles in time.

### 4.3. Curved Driving Experiments

JT/T883-2014 only regulates the straight driving experiments but does not request or give advice to curved driving experiments. However, to further validate the superiority of our system, we conduct curved driving experiments and compare its accuracy to a commercial MMWR, Aptiv (Electronically Scanning RADAR) ESR 2.5, which is used in CWS. The MMWR measures the relative distance, relative azimuth, and relative velocity. Table 10 shows the accuracy of Aptiv ESR 2.5 according to its datasheet. *ρ*, *θ*, and *v* represent the relative distance, azimuth angle, and velocity, respectively. The MMWR has two working modes, middle-distance mode and long-distance mode, and the accuracies are different.

**Figure 21:**
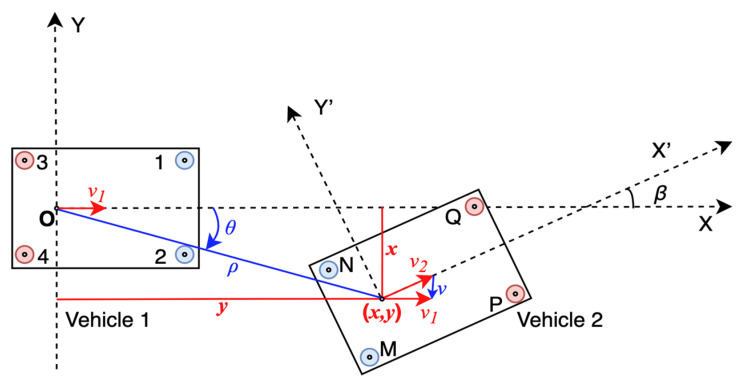
The transformation from Cartesian coordinates to polar coordinates.

In order to facilitate comparison, the curved experiments are also divided into a middle-distance experiment under vehicle distances within 50 m and a long-distance experiment under vehicle distances within 100 m. The estimated values of the relative position [*x*, *y*] are converted to the polar coordinate [*ρ*, *θ*], and the velocities of the two vehicles [*v*_1_, *v*_2_] are converted to the relative velocity *v*, as shown in Figure 21. In addition, the relative orientation *β* cannot be measured by MMWR directly.

#### 4.3.1. Middle-Distance Experiments

The proposed CWS and MMWR are all dynamic systems, so the vehicle distance is not kept to a constant value but changes in the experiment. During the middle-distance experiment, the vehicle distance changes between 10 and 50 m. In our system, the vehicle distance represents the distance between the real axle centers of the two vehicles, so it cannot be zero.

#### 4.3.2. Long-Distance Experiments

During the long-distance experiment, the vehicle distances change between 10 and 100 m.

#### 4.3.3. Results Analysis of the Curved Experiments

**Figure 22:**
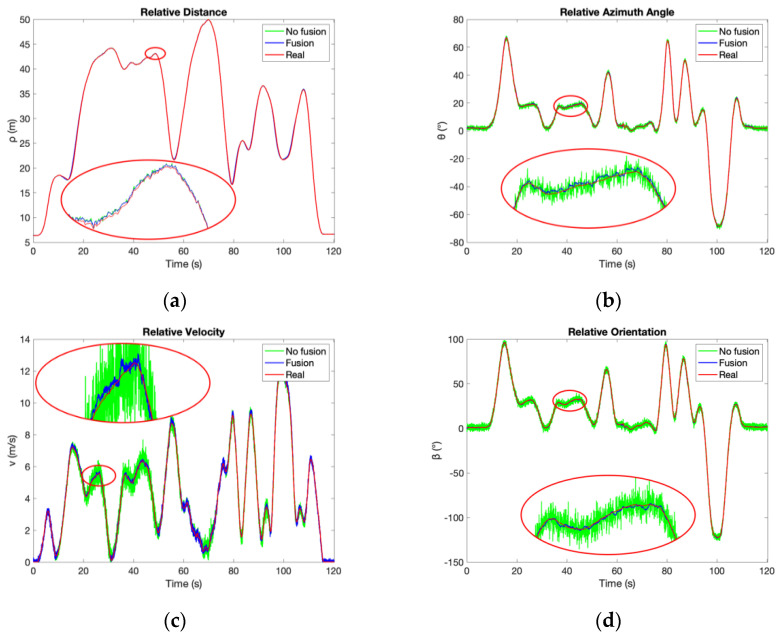
The results of the middle-distance experiments. (**a**) Relative distance; (**b**) Relative azimuth angle; (**c**) Relative velocity; (**d**) Relative orientation.

**Figure 23:**
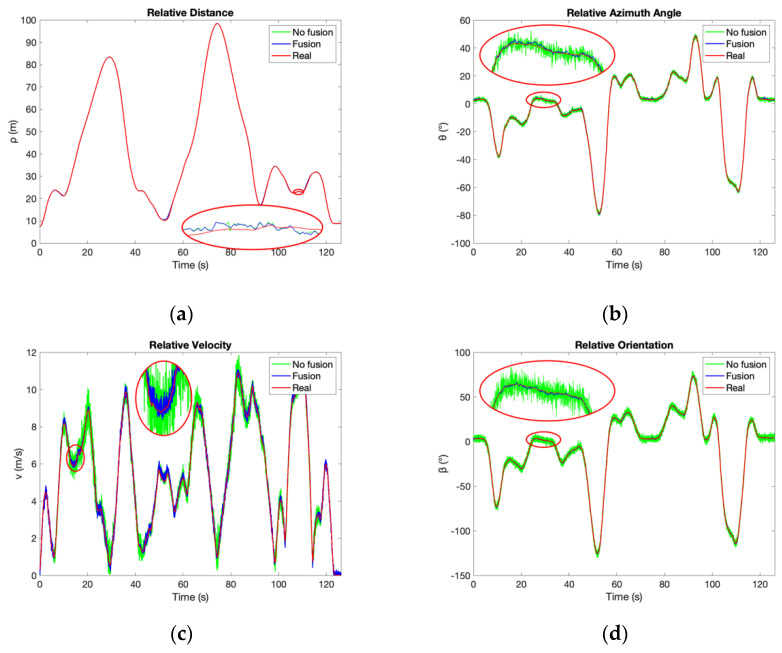
The results of the long-distance experiments. (**a**) Relative distance; (**b**) Relative azimuth angle; (**c**) Relative velocity; (**d**) Relative orientation.

According to Figure 22 and Figure 23, the accuracy of the proposed system improves significantly after fusion, which reaches the same conclusion as the simulation results shown in Figure 12. The relative position is described as Cartesian coordinate [*x*, *y*] in the simulation but as polar coordinate [*ρ*, *θ*] in the experiments. Both x and y improve after fusion as shown in Table 2, whereas only *θ* without *ρ* improves after fusion according to Table 11 and Table 12. That is because the accuracy improvement of *θ* can contribute to better accuracy of both *x* and *y*, as Figure 21. Therefore, the experimental results are consistent with the simulation. The comparison result of the proposed system and the MMWR is shown in Table 13, which combines Table 11 and Table 12 with Table 10.

The distance accuracy of the proposed system is always much better than the MMWR, no matter with or without fusion. The azimuth accuracy without fusion is about 0.76° in both experiments, which is better than the middle-distance MMWR but is inferior to the long-distance MMWR. However, velocity accuracy without fusion is worse than the MMWR in both modes. As for the fusion system, the accuracy of relative distance and azimuth performs significantly better than the MMWR, and the relative velocity accuracy also improves to a similar level as the MMWR in both middle and long-distance modes. Table 14 shows the accuracy enhanced rates of the proposed system to the MMWR.

In addition, the proposed system can provide the relative orientation, which is not available directly in the MMWR system.

## 5. Conclusions

In this paper, we proposed a CWS combining UWB and DR. An improved relative positioning/directing algorithm based on UWB is presented, and a DR model based on the speeds of the rear wheels is established. Then, a fusion algorithm using EKF is proposed to improve the accuracy of relative position, orientation, and velocity. Afterwards, the advantage of the proposed system is preliminarily verified by simulation. Finally, experiments are conducted to further validate the performance of our system, and the experiment results are compared to a commercial MMWR used in CWS. The main conclusions are summarized as follows:

- The proposed relative positioning/directing algorithm with an additional distance constraint significantly improves the relative positioning/directing accuracy, especially the directing accuracy, as shown in Figure 9 and Table 1.
- The fusion method significantly improves the relative positioning/directing accuracy and slightly improves the velocity accuracy according to the simulation and experiment results.
- The proposed CWS passes the regulated tests in JT/T883-2014 published by MOT, which proves the feasibility of the proposed system.
- In middle-distance mode up to 50 m, compared to the MMWR, the proposed system improves the relative positioning/directing accuracy by 44%, 69%, and 8%, respectively, in the relative distance, azimuth angle, and velocity. As for in long-distance mode, the enhanced rate is 66% and 38%, respectively, for the relative distance and azimuth angle. The relative velocity accuracy of the proposed system is similar to the MMWR.
- In both middle and long-distance modes, the proposed system can provide relative orientations with errors no more than 0.4° RMSE, which is not available directly in MMWR systems, but it is very beneficial to the CWS.

The inadequacy of the proposed system is the velocity accuracy. Although it performs at the same level as MMWR in terms of velocity accuracy, it can be further improved. To facilitate comparison of the proposed system and the MMWR, the velocity data of the experiments are shown as relative velocity. We also analyze the accuracy of absolute velocities of two vehicles. In the middle-distance experiment, $RMSE_{v_{1}}=0.16\;m/s$ and $RMSE_{v_{2}}=0.13\;m/s$, and in the long-distance experiment, $RMSE_{v_{1}}=0.16\;m/s$ and $RMSE_{v_{2}}=0.16\;m/s$. Both of them are inferior to the simulation results. It is because the DR system is established based on a theoretical Ackerman steering model, which ignores the stiffness of suspensions and tires. In the actual situation, vehicle dynamic parameters such as side-slip angles will also affect the precision of the algorithm. Therefore, our research direction in the future is the system with a more accurate vehicle dynamic model and with more sensors integrated such as IMU and GPS.

## Figures and Tables

**Table 1:** RMSE of the algorithm with and without *d*_5_.

Algorithm	*RMSE_x_* (m)	*RMSE_y_* (m)	*RMSE_β_* (°)
With *d*_5_	0.70	0.73	46.29
Without *d*_5_	0.21	0.58	2.42

**Table 2:** RMSE of position and orientation estimated by UWB and UWB/DR.

Algorithm	*RMSE_x_* (m)	*RMSE_y_* (m)	*RMSE_β_* (°)
UWB	0.21	0.58	2.42
UWB + DR (EKF)	0.06	0.17	0.83

**Table 3:** RMSE of velocities and yaw rates estimated by DR and UWB/DR.

Algorithm	$RMSE_{\omega_{1}}\;({}^{\circ}/s)$	$RMSE_{\omega_{2}}\;({}^{\circ}/s)$	$RMSE_{v_{1}}\;(m/s)$	$RMSE_{v_{2}}\;(m/s)$
DR	8.92	8.71	0.14	0.15
UWB + DR (EKF)	5.07	4.60	0.12	0.08

**Table 4:** Ranges of parameters in TTC simulation.

Parameters	Range
***v*_1_&*v*_2_** (**km/h**)	0~75
***x*** (**m**)	−200~200
***y*** (**m**)	−15~15
***β*** (**°**)	0~360

**Table 5:** Collision warning evaluation in rear-end scenarios.

Evaluation	Quantity
Failed	0
Correct	194
False	2

**Table 6:** Collision warning evaluation in random scenarios.

Evaluation	Quantity
Failed	0
Correct	10,596
False	227

**Table 7:** Results of Test 1.

	1	2	3	4	5	6	7
TTC(CWS)	2.9987	2.9907	2.9759	2.9814	2.9729	2.9722	2.9799
TTC(Real)	3.0047	3.0069	2.9925	2.9963	2.9902	3.0136	3.0219
Evaluation	Pass	Pass	Pass	Pass	Pass	Pass	Pass

**Table 8:** Results of Test 2.

	1	2	3	4	5	6	7
TTC(CWS)	2.9863	2.9810	2.9987	2.9804	2.9954	2.9899	2.9673
TTC(Real)	3.0423	3.1166	3.0245	3.0354	3.1283	3.1269	3.0226
Evaluation	Pass	Pass	Pass	Pass	Pass	Pass	Pass

**Table 9:** Results of Test 3.

	1	2	3	4	5	6	7
TTC(CWS)	2.9782	2.9905	2.9947	2.9789	2.9942	2.8623	2.8958
TTC(Real)	2.7560	2.8110	2.7877	2.6851	2.6511	2.5831	2.8975
Evaluation	Pass	Pass	Pass	Pass	Pass	Pass	Pass

**Table 10:** The accuracy of the MMWR.

Mode	Coverage (m)	*RMSE_ρ_* (m)	*RMSE_θ_* (°)	*RMSE_v_* (m/s)
Middle Distance	50	0.25	1	0.12
Long Distance	100	0.5	0.5	0.12

**Table 11:** The accuracy of the MMWR.

Mode	*RMSE_ρ_* (m)	*RMSE_θ_* (°)	*RMSE_v_* (m/s)	*RMSE_β_* (°)
No Fusion	0.14	0.76	0.22	1.84
Fusion	0.14	0.31	0.11	0.39

**Table 12:** The accuracy of the MMWR.

Mode	*RMSE_ρ_* (m)	*RMSE_θ_* (°)	*RMSE_v_* (m/s)	*RMSE_β_* (°)
No Fusion	0.18	0.77	0.22	1.86
Fusion	0.17	0.31	0.12	0.40

**Table 13:** Accuracy comparison of the proposed system and the MMWR.

Mode	System	*RMSE_ρ_* (m)	*RMSE_θ_* (°)	*RMSE_v_* (m/s)	*RMSE_β_* (°)
Middle Distance	MMWR	0.25	1	0.12	None
	Proposed System (No Fusion)	0.14	0.76	0.22	1.84
	Proposed System (Fusion)	0.14	0.31	0.11	0.39
Long Distance	MMWR	0.5	0.5	0.12	None
	Proposed System (No Fusion)	0.18	0.77	0.24	1.86
	Proposed System (Fusion)	0.17	0.31	0.12	0.40

**Table 14:** Enhanced rate of the proposed system to the MMWR.

Mode	*RMSE_ρ_* (m)	*RMSE_θ_* (°)	*RMSE_v_* (m/s)
Middle Distance	44%	69%	8%
Long Distance	66%	38%	0%
